# Recognition of the Impulse of Love at First Sight Based on Electrocardiograph Signal

**DOI:** 10.1155/2021/6631616

**Published:** 2021-03-22

**Authors:** Jin Zhang, Guangjie Yuan, Huan Lu, Guangyuan Liu

**Affiliations:** ^1^College of Electronic and Information Engineering, Southwest University, Chongqing, China; ^2^Institute of Affective Computing and Information Processing, Southwest University, Chongqing, China; ^3^Chongqing Key Laboratory of Nonlinear Circuits and Intelligent Information Processing, Southwest University, Chongqing, China

## Abstract

The impulse of love at first sight (ILFS) is a well known but rarely studied phenomenon. Despite the privacy of these emotions, knowing how attractive one finds a partner may be beneficial for building a future relationship in an open society, where partners are accepting each other. Therefore, this study adopted the electrocardiograph (ECG) signal collection method, which has been widely used in wearable devices, to collect signals and conduct corresponding recognition analysis. First, we used photos to induce ILFS and obtained ECG signals from 46 healthy students (24 women and 22 men) in a laboratory. Second, we extracted the time- and frequency-domain features of the ECG signals and performed a nonlinear analysis. We subsequently used a feature selection algorithm and a set of classifiers to classify the features. Combined with the sequence floating forward selection and random forest algorithms, the identification accuracy of the ILFS was 69.07%. The sensitivity, specificity, F1, and area under the curve of the other parameters were all greater than 0.6. The classification of ECG signals according to their characteristics demonstrated that the signals could be recognized. Through the information provided by the ECG signals, it can be determined whether the participant possesses the desire to fall in love, helping to determine the right partner in the fastest time; this is conducive to establishing a romantic relationship.

## 1. Introduction

The impulse of love at first sight (ILFS) is a significant initial attraction [1], that is, a strong desire to relate with another person, and is a complex phenomenon that includes evaluation, appreciation, and subjective experience of physiological changes. ILFS can be observed in many literary and artistic works. In real life, the concept of ILFS is accepted by most people. For example, approximately one-third of westerners report that they have experienced ILFS [2]. Moreover, studies have observed that ILFS can affect relationships [3, 4]. A relationship between couples involving the ILFS is more passionate, causing the relationship to be more stable and satisfying [5]. Vico et al. [6] observed that, in the presence of a favorite face, heart rate and skin conductance activity increase, along with valence and arousal and a reduction in dominance evaluation. Fisher [7] found that the psychological responses of ILFS include excitement, increased energy, tremor, rapid heartbeats, and shortness of breath. Nevertheless, almost no research has been conducted on recognizing the ILFS. In general, the ILFS is a type of emotional state that can be studied by referring to previous methods of emotion recognition.

In recent years, physiological signals, such as electroencephalograms [8], electrocardiograms (ECGs) [9–11], electromyography [12], photoplethysmography [13], galvanic skin [14] response, and respiration, have been widely applied in the field of emotion recognition. On the one hand, behavioral data (such as facial expressions and body postures) and voice data are easily manipulated by subjective consciousness [15]; on the other hand, physiological signals are real-time and continuous signals that can be used to better analyze the expression and conversion between different emotional states. Among these physiological signals, emotion recognition using ECG signals has become an important topic in the field of emotion computing. First, ECG signal-derived features, such as heart rate (HR) and heart rate variability (HRV), have been observed as reliable physiological indicators of emotion recognition [16, 17]. For example, Kreibig [18] demonstrated that happiness results in a reduction in HRV while joy and entertainment increase HRV. Research by Lichtenstein et al. [19] indicated that there are significant differences in HRV corresponding to anger and happiness, anger and satisfaction, and sadness and happiness. In addition, Rainville [20] demonstrated that HR and HRV characteristics can be used to distinguish four emotions: anger, fear, happiness, and sadness. Second, ECG signals have been widely used for emotion recognition owing to the low cost, portability, wearability, and wireless advantages of ECG devices. Karthikeyan et al. [21] distinguished between relaxed and stressed states using ECG signals and achieved a classification accuracy of 94.6%. Guo et al. [22] extracted HRV features from ECG signals and used support vector machines (SVMs) to classify different emotional states. The results demonstrated that the two emotional states (positive/negative) attained 71.4% accuracy. Castaldo et al. [23] evaluated the potential of stress detection using an ultra-short-term HRV analysis. The experimental results showed that the sensitivity, specificity, and accuracy of classification surpassed 60% using ultra-short-term HRV features for classification. Hsu et al. [15] proposed an ECG-based automatic emotion recognition algorithm. The classification accuracy of positive/negative valence, high/low arousal, and three types of emotions (joy, sadness, and peacefulness) using a least-squares SVM were 82.78%, 72.91%, and 61.25%, respectively.

In addition, in the study of emotion recognition, pictures [24], music [25], movies [26], and text [27] are frequently used to elicit emotions. This study examines ILFS when two people meet each other. Conducting a speed dating scenario with hundreds of participants in a laboratory environment is not feasible. Moreover, the ILFS studied in this study can be generated in a very short time. Therefore, in this study, we used images to induce ILFS and used ECG signals to classify and recognize ILFS. Also, we designed an accurate experiment to collect ECG signals from participants during the viewing period. Subsequently, we developed an automatic ILFS recognition algorithm to detect the *R* wave, generate important features related to the ILFS, and effectively identify the ILFS.

The remainder of this paper is organized as follows: Section 2 describes the experimental setup and protocol. The proposed ECG-based ILFS recognition algorithm is introduced in Section 3.  Section 4 presents the results and corresponding discussion.  Section 5 presents the conclusions of this study.

## 2. Experimental Setup

### 2.1. Experiment Material

In this study, various factors were comprehensively considered to select photos as the stimulus material; 800 photos of smiling men and women were purchased and downloaded from a photo website. Subsequently, these photos were cropped into bust photos with uniform properties, for example, size, brightness, and resolution.

Unified processed pictures were scored and formal test materials were selected. Psychologists have shown that facial attractiveness is strongly linked to ILFS [28, 29]. Every time the unit of attraction increases by one level, the likelihood of the ILFS will increase by a factor of nine. Therefore, 60 college students (30 men and 30 women) with no colorblindness or physical/mental health were recruited from Southwestern University to evaluate the facial attractiveness of photos of the opposite sex and were asked to subjectively evaluate facial attractiveness on a scale of 1 (not at all) to 9 (extremely). We then selected 240 male and 240 female photos from those evaluated as material that induced ILFS (high attraction: average: low attraction ≈0.25 : 0.6 : 0.15).

### 2.2. Participants

The researchers recruited 46 healthy Southwestern University students (24 women and 22 men; mean age, 19.7 ± 1.6 years). The participants were required to abstain from vigorous exercise for 2 h before the experiment to avoid a rapid heart rate, which would affect the experimental data and results. However, owing to equipment problems, the data of the three students were not used.

All participants provided written informed consent. Before data collection, all methods were approved by the Human Ethics Research Committee of Southwestern University.

### 2.3. Experimental Context

**Figure 1:**
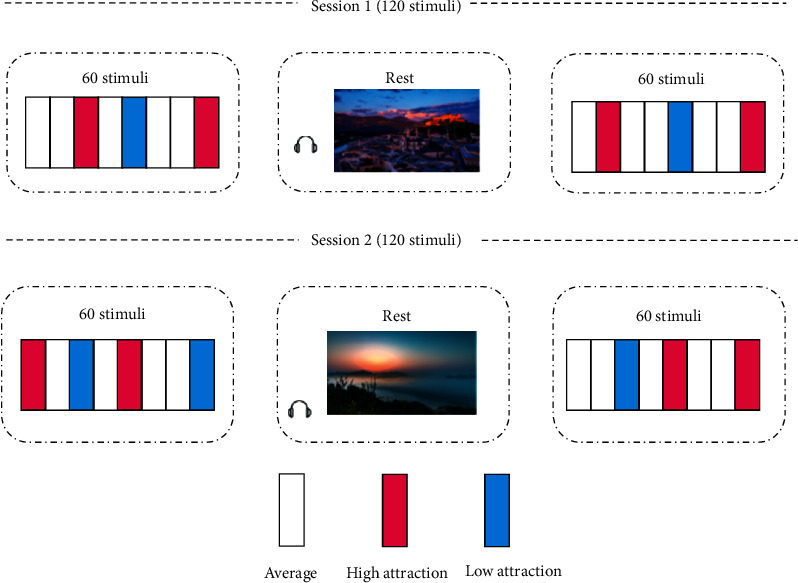
Experimental paradigm.

This experiment was divided into two sessions (two sessions were performed at least one day apart). Each session contained 120 stimuli. Each session had two blocks and each block contained 60 stimulus materials. In the experiment, the presentation time of each stimulus material was 10 s and the participants were evaluated according to their emotional state. After each block, a neutral landscape and a piece of light music were presented for 4 min. The experimental paradigm is illustrated in Figure 1.

At the beginning of the experiment, the subjects sat quietly in a chair and their bodies were in a state of natural relaxation. The corresponding picture stimulus materials were then presented according to the written emotion-induced experimental paradigm to induce the ILFS. After the subjects watched the stimulus materials, they performed the emotion induction evaluation and subjectively reported the ILFS induction intensity for each stimulus material, in the range of 0 (none) to 3 (extreme). ECG signals were collected using an MP150 system and the sampling frequency was set to 1000 Hz. After the experiment was completed, the subjects were asked to look at the pictures again and subjectively report their arousal, valence, dominance, and attraction, in the range of 1 to 7. The self-report rating scale used here was a Likert table [30].

## 3. Methodology

**Figure 2:**
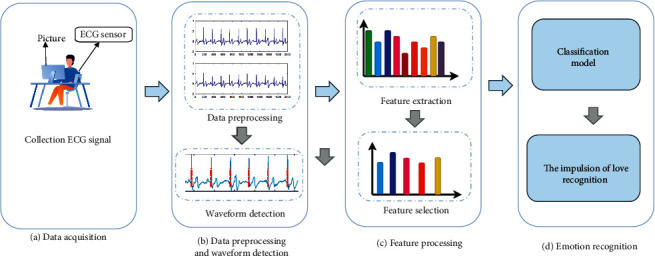
Frame diagram of state recognition of ILFS.

In summary, ECG signals were recorded for 46 participants observing 240 pictures of the opposite sex. Subsequently, the ECG signals were preprocessed to remove the interference and noise. After noise removal, feature extraction was performed on the signals and the time domain, frequency, and nonlinear features of the ECG signal were extracted. After extracting some statistical features (indices), we employed a feature selection algorithm to reduce the feature dimensions, thereby reducing the computational cost. Finally, different classifiers are used for sentiment classification. The frame diagram of the state recognition of the ILFS is shown in Figure 2.

### 3.1. Preprocessing

Before preprocessing, the ECG signal was downsampled to 200 Hz.

The ECG signal is a nonstationary weak signal that easily receives interference from itself and the outside environment; this interference and noise may conceal useful information. Before feature extraction, the original ECG signal must be preprocessed. ECG frequently includes baseline drift below 1 Hz, power frequency interference at 50 Hz, and electromyographic interference. During preprocessing, a discrete wavelet transform—a common method for removing noise [31]—was used. The original ECG signal was scaled using a discrete wavelet transform, the approximate coefficients and detail coefficients of each layer were extracted, and the soft threshold function was used to process the detail coefficients. Subsequently, a pure ECG signal was reconstructed.

The noise-removed ECG signal was divided into 10 s time signals when the stimulus material appeared as the starting point. Subsequently, the Pan–Tompkins peak detection algorithm was used to locate the R-wave peak to obtain the RR interval [32]. HRV parameters can be obtained through feature extraction of the RR interval. HRV is a reliable marker of activity in the autonomic nervous system and reflects the time change of a continuous heartbeat [33].

### 3.2. Feature Extraction

In this study, twenty-five features were extracted from the ECG signals, including the HRV time domain, frequency domain, and nonlinear characteristics; details of the feature information are presented in Table 1.

### 3.3. Construction of ILFS and Non-ILFS Datasets

Before constructing the datasets, we first removed the abnormal data, which would have affected the classification results.

The median absolute deviation (MAD) algorithm can effectively remove outliers from the data [34]. The MAD and outlier removal methods are shown in the following equations, respectively:

(1) $$\begin{array}{c} \text{MAD}={\text{median}}_{i}\left(\left|x_{i}-{\text{median}}_{j}\left(x_{j}\right)\right|\right), \end{array}$$

(2) $$\begin{array}{c} \left\{\begin{array}{c} x_{i}\leq \text{median}\left(x_{i}\right)-5\times \text{MAD},\\ x_{i}\geq \text{median}\left(x_{i}\right)+5\times \text{MAD}, \end{array}\right) \end{array}$$

where *x*_*j*_ is one of the *n* sample values and median_*i*_ is the median of the series.

By summarizing previous studies on the ILFS [6, 7, 35], we consider that the ILFS exhibits the characteristics of high arousal, high price, high attractiveness, and high dominance. Therefore, combining the two evaluations in the experiment, the data with high arousal, high price, high attractiveness, and high dominance were screened from the ILFS data (data with a level of 1 for the ILFS were not used) as the dataset of ILFS states. In addition, for the non-ILFS data, data with low arousal, low valence, low attractiveness, and low dominance were selected from the data without the ILFS as the non-ILFS dataset.

### 3.4. Feature Selection

A feature selection algorithm can remove redundant features and reduce the quantity of data, thereby improving the classification accuracy and significantly reducing the computational cost [36]. We thus selected the sequence floating forward selection (SFFS) algorithm. The SFFS algorithm selects an optimal feature subset as the classification input and can solve the local optimization problem of the feature set to a certain extent [37].

SFFS combines sequential forward selection (SFS) and sequential backward selection (SBS) algorithms. The SFFS has three parts: insertion, conditional exclusion, and termination.

First, let *F*_*k*_={*f*_*i*_ : 1 ≤ *i* ≤ *k*} be a feature subset composed of *k* features selected from the original feature set *Y*={*y*_*i*_ : 1 ≤ *i* ≤ *n*}, where *n* is the total number of features. The evaluation function of the optimal feature subset was *J*(·).

 
*Step 1.* Inclusion: beginning from the empty set *F*=∅, use the SFS method to select the most important feature *f*^+^ from {*Y* − *F*_*k*_} and *F*_*k*_ to form a new feature subset *F*_*k*+1_, and *F*_*k*+1_=*F*_*k*+1_+*f*^+^. Set *k* = *k* + 1 to execute Step 2.
 
*Step 2.* Conditional exclusions determine the most important feature (*f*^−^) from *F*_*k*+1_, if *f*^−^ is the most important feature in *F*_*k*+1_, and *J*(*F*_*k*+1_ − *f*^−^) > *J*(*F*_*k*_); delete *f*^−^ from *F*_*k*+1_ to form a new feature subset *F*_*k*_′ and *F*_*k*_′=*F*_*k*+1_ − *f*^−^. Thereafter, Step 3 is performed. In addition, if *J*(*F*_*k*+1_ − *f*^−^) < *J*(*F*_*k*_), return to Step 1.
 
*Step 3.* Termination. Set *k* = *k* – 1; if *k* is equal to the expected number of features, stop. Otherwise, set *F*_*k*_=*F*_*k*_′, *J*(*F*_*k*_)=*J*(*F*_*k*_′), and return to Step 1.

In this study, two nested 10-fold cross-validation schemes were used to obtain reliable model estimates for feature selection and model training [35]. The best feature subset is selected in the inner loop. In the outer loop, using the selected best feature subset, the classifier was evaluated using 10-fold cross-validation.

## 4. Results and Discussion

This section presents a series of results (feature analysis and classification results) to evaluate the effectiveness of the proposed approach. In addition, the results were comprehensively discussed.

### 4.1. Feature Analysis

**Figure 3:**
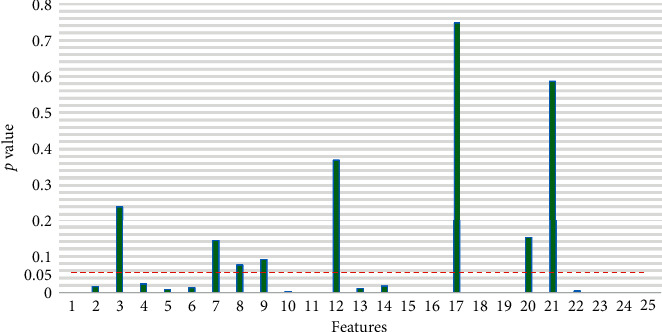
Characteristic *p* value between the ILFS and non-ILFS.

We evaluated whether the characteristics of the ECG are shown in Table 1; the characteristics of the ILFS data sample, and the characteristics of the non-ILFS data were significantly different. The Wilcoxon signed-rank test is the most extensive nonparametric rank-sum test method for two independent groups [38]. A *p* value of less than 0.05 indicates that a significant difference exists between the ILFS and non-ILFS. As Figure 3 illustrates, the results of the Wilcoxon test indicated that the differences in ILFS and non-ILFS status for features #3, #7, #8, #9, #12, #17, #20, and #21 were insignificant. Consistent with the results in [6, 7], the Wilcoxon test results of feature #11 indicate that the ILFS state exhibits a higher heart rate.

Although some features are not significantly different between the ILFS and non-ILFS states, the classification performance can be significantly improved when used in combination with other features [39]. Therefore, we used 25 heartbeat feature vectors to represent each sample in the ILFS and non-ILFS state datasets.

**Figure 4:**
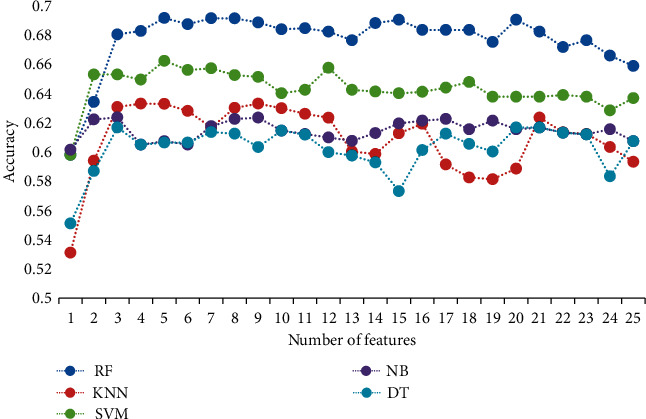
Results of the accuracy of five classifiers for selecting different numbers of features.

Feature selection involves selecting the fewest features without affecting the classification effect. Thus, in this study, 10-fold cross-validation schemes based on the SFFS algorithm were used for feature selection. The number of features was changed from 1 to 25 for training and the best feature subset was selected.  Figure 4 shows the accuracy of the five classifiers for selecting different numbers of features. The features corresponding to the maximum accuracy of the different classifiers were used as the optimal feature subset of the classifier.

Table 2 lists the best feature subsets of the different classifiers. It can be seen from Table 2 that feature #1 is one of the best performing features in each classifier and feature #1 is reduced in the ILFS state. Consistent with the literature results [7, 35], ILFS produces physiological reactions, for example, excitement, a rapid heartbeat, an increased heart rate in the excited state, and a reduced average RR interval.

### 4.2. Classification Result

**Figure 5:**
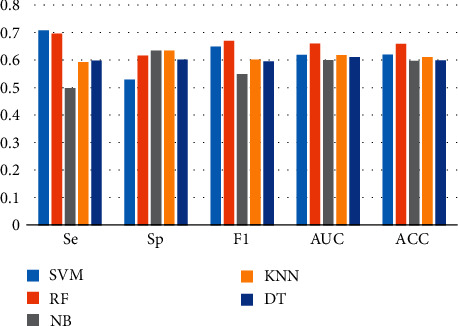
Classification performance of the five classifiers without feature selection.

In this research, the ILFS and non-ILFS samples were classified using a set of widely used classifiers, such as SVM, random forest (RF), and naive Bayes (NB). In addition, sensitivity (Se), specificity (Sp), F1-score (F1), area under the curve (AUC), accuracy (ACC), and other parameters were used to evaluate the performance of the classification scheme.  Table 3 and Figure 5 present the classification performance of five classifiers without feature selection for ECG signals. Among these classifiers, RF exhibits the best classification accuracy, with a result of 66.04%. Other classifiers recognized the ILFS and their classification accuracy was approximately 60%. The parameters Se, Sp, F1, and AUC of the classifier were all approximately 0.6.

**Figure 6:**
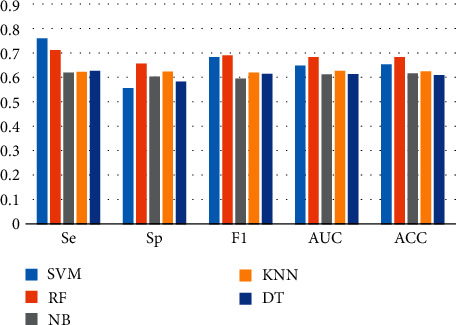
Classification performance after feature selection.

During the analysis presented in the previous section, the optimal feature subset of the classifier was obtained based on the SFFS algorithm and the optimal feature subset was used to evaluate the classifier using 10-fold cross-validation.  Table 4 and Figure 6 show the classification performance of the five classifiers after feature selection. The results demonstrate that the highest accuracy rate of 69.07% is obtained for the classifier RF and features #1, #3, #8, #12, and #24 constitute the best feature subset; the parameters Se, Sp, F1, and AUC of the classifier RF were better than those of the other classifiers; however, the parameters of all classifiers are greater than 0.6, indicating that ILFS can be classified and recognized.

**Figure 7:**
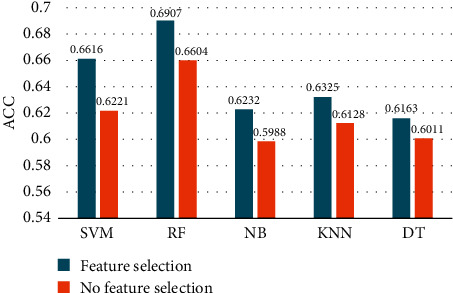
Comparison of the classification accuracy of different classifiers with no feature selection and feature selection.

Figure 7 shows that after using feature selection, the classification effect of 5 classifiers is improved. In addition, it can be seen that the feature selection method (combined with the RF classifier) is optimal for identifying ILFS emotions, compared to other machine learning algorithms.

In previous studies, few researchers have examined the mapping pattern between the ILFS and physiological signals. Therefore, in this paper, a study on the classification and recognition of the ILFS based on ECG signals is proposed, that is, the use of an ECG signal to identify whether someone is in a state of ILFS. The best classification accuracy rate was 66.04% for all signal characteristics. With the SFFS feature selection algorithm, the best classification accuracy increased to 69.03%. The experimental results show that ILFS can be classified and identified based on ECG signals; however, the recognition and classification of the ILFS based on ECG signals are not very accurate. The following may be factors that affect the classification of ILFS:

1. The recognition effect of the ILFS is related to the classifier used. Selecting a more advanced classification algorithm can improve the classification effect.
2. The ILFS is highly related to the subjects' aesthetic preferences and the emotional intensity induced by the selected stimulus photos is insufficient.
3. The ILFS is a complex emotional state. Accurately reflecting the changes in the ILFS using only an ECG signal is difficult.

Therefore, in future research, for better classification and recognition of the ILFS, it is necessary to (1) determine a more advanced classification algorithm, (2) use different stimuli (e.g., video) to induce a higher intensity of the ILFS, and (3) use a variety of physiological signals.

## 5. Conclusions

This study attempted to identify the ILFS based on ECG signals. Our research demonstrated that the ILFS is separable. Based on the recognition of the ILFS using the ECG, through the information provided by the physiological signal, people can determine whether they have the ILFS. Determining the right partner in the fastest time is conducive to establishing a relationship. Moreover, owing to the advantages of low cost, portability, and wearable devices, the ECG signal-based ILFS recognition algorithm can be combined with wearable devices, which can better match the cardiac target in specific scenarios, online or offline.

## Figures and Tables

**Table 1:** ECG characteristic description.

Number	Symbol	Feature description
Time-domain features		
1	Mean_RR	Mean of RR intervals
2	CVRR	The coefficient of variance of RR intervals
3	SDRR	Standard deviation of RR intervals
4	RMSSD	Root mean square of successive differences of RR intervals
5	MSD	Mean of the absolute values of the first differences of RR intervals
6	SDSD	Standard deviation of successive differences of RR intervals
7	NN50	Number of interval differences of successive RR intervals greater than 50 ms
8	PNN50	Corresponding percentage of RR50
9	NN20	Number of interval differences of successive RR intervals greater than 20 ms
10	PNN20	Corresponding percentage of RR20
11	Mean_HR	Average heart rate
12	QD	Quartile deviation of RR intervals

Nonlinear features		
13	SD1	Standard deviation for *T* direction in Poincare plot
14	SD2	Standard deviation for *L* direction in Poincare plot
15	SD1_SD2	SD1/SD2
16	CSI	Cardiac sympathetic index
17	CVI	Cardiac vagal index
18	modified_CSI	Modified CSI
19	LZC	LZ complexity

Frequency-features		
20	TP	Power of range 0.04–0.4 Hz of the PSD of RR intervals
21	LF	Power of range 0.04–0.15 Hz of the PSD of RR intervals
22	HF	Power of range 0.15–0.4 Hz of the PSD of RR intervals
23	LF/HF	Proportion of LF to HF
24	nLFP	Proportion of LF to LF + HF
25	nHFP	Proportion of HF to LF + HF

**Table 2:** The best feature subsets of five classifiers.

Classifier	Selected features
SVM	1, 3, 8, 15, 14
RF	1, 3, 8, 12, 24
NB	1, 2, 11
KNN	4, 7, 8, 12
DT	1, 8, 24

**Table 3:** Classification performance of the five classifiers without feature selection.

Classifier	Se	Sp	F1	AUC	ACC
SVM	0.7103	0.5305	0.6512	0.6209	0.6221
RF	**0.6984**	**0.6186**	**0.6717**	**0.6616**	**0.6604**
NB	0.5	0.6363	0.5513	0.6017	0.5988
KNN	0.5949	0.6363	0.6037	0.62	0.6128
DT	0.6001	0.6037	0.5977	0.6122	0.6011

SVM: support vector machine; RF: random forest; NB: naive bayes; KNN: K-nearest neighbor; DT: decision tree.

**Table 4:** Classification performance of five classifiers after feature selection.

Classifier	Se	Sp	F1	AUC	ACC
SVM	0.7684	0.5631	0.6908	0.656	0.6616
RF	**0.7194**	**0.6635**	**0.6984**	**0.6903**	**0.6907**
NB	0.627	0.6101	0.6021	0.6194	0.6232
KNN	0.6307	0.631	0.627	0.634	0.6325
DT	0.6343	0.5897	0.6216	0.6209	0.6163

## Data Availability

The data used to support the findings of this study are available from the corresponding author upon request.
